# Genotyping of enteroviruses isolated in Kenya from pediatric patients using partial VP1 region

**DOI:** 10.1186/s40064-016-1834-0

**Published:** 2016-02-24

**Authors:** Silvanos M. Opanda, Fred Wamunyokoli, Samoel Khamadi, Rodney Coldren, Wallace D. Bulimo

**Affiliations:** Department of Emerging Infectious Diseases (DEID), United States Army Medical Research Directorate-Kenya, P.O. Box 606-00621, Nairobi, Kenya; The Kenya Medical Research Institute (KEMRI), Nairobi, Kenya; Department of Biochemistry, Jomo Kenyatta University of Agriculture and Technology (JKUAT), Nairobi, Kenya; College of Health Sciences (COHES), Jomo Kenyatta University of Agriculture and Technology (JKUAT), Nairobi, Kenya; Department of Biochemistry, School of Medicine, University of Nairobi, Nairobi, Kenya

**Keywords:** Genotyping, Human enterovirus, VP1 gene, Kenya

## Abstract

Enteroviruses (EV) are responsible for a wide range of clinical diseases in humans. Though studied broadly in several regions of the world, the genetic diversity of human enteroviruses (HEV) circulating in the sub-Saharan Africa remains under-documented. In the current study, we molecularly typed 61 HEV strains isolated in Kenya between 2008 and 2011 targeting the 3′-end of the VP1 gene. Viral RNA was extracted from the archived isolates and part of the VP1 gene amplified by RT-PCR, followed by sequence analysis. Twenty-two different EV types were detected. Majority (72.0 %) of these belonged to Enterovirus B species followed by Enterovirus D (21.3 %) and Enterovirus A (6.5 %). The most frequently detected types were Enterovirus-D68 (EV-D68), followed by Coxsackievirus B2 (CV-B2), CV-B1, CV-B4 and CV-B3. Phylogenetic analyses of these viruses revealed that Kenyan CV-B1 isolates were segregated among sequences of global CV-B1 strains. Conversely, the Kenyan CV-B2, CV-B3, CV-B4 and EV-D68 strains generally grouped together with those detected from other countries. Notably, the Kenyan EV-D68 strains largely clustered with sequences of global strains obtained between 2008 and 2010 than those circulating in recent years. Overall, our results indicate that HEV strains belonging to Enterovirus D and Enterovirus B species pre-dominantly circulated and played a significant role in pediatric respiratory infection in Kenya, during the study period. The Kenyan CV-B1 strains were genetically divergent from those circulating in other countries. Phylogenetic clustering of Kenyan EV-D68 strains with sequences of global strains circulating between 2008 and 2010 than those obtained in recent years suggests a high genomic variability associated with the surface protein encoding VP1 gene in these enteroviruses.

## Background

Enteroviruses (EV), family *Picornaviridae* and genus *Enterovirus*, are common etiologic agents of diseases affecting millions of people worldwide every year (Jacques et al. 2008; Oberste et al. 2003). The disease manifestations range from asymptomatic to a wide variety of acute diseases, including mild respiratory illnesses, hand, foot and mouth disease, aseptic meningitis, encephalitis, myocarditis, and neonatal sepsis-like diseases (Tan et al. 2011; Khetsuriani et al. 2006; Kiang et al. 2009; Harvala et al. 2014; Chiang et al. 2012; Linsuwanon et al. 2012; Kumar et al. 2013). They are classified into twelve species based on molecular characteristics and biological properties (Kumar et al. 2013; Nasri et al. 2007; Hu et al. 2011; Pallansch and Roos 2007). Seven of these species, namely Enterovirus A, Enterovirus B, Enterovirus C, Enterovirus D, Rhinovirus A, B and C affect humans (Adams et al. 2014, 2015). Enterovirus A species comprises Coxsackievirus A2 (CV-A2), CV-A3, CV-A4, CV-A5, CV-A6, CV-A7, CV-A8, CV-A10, CV-A12, CV-A14, CV-A16, enterovirus A71 (EV-A71), EV-A76, EV-A89, EV-A90, EV-A91, EV-A92, EV-A114, EV-A119, EV-A120 and EV-A121 (Oberste et al. 2004, 2005; Pöyry et al. 1994; Brown and Pallansch 1995; Adams et al. 2013; King et al. 2012). Enterovirus B species includes Coxsackievirus B1 (CV-B1), CV-B2, CV-B3, CV-B4, CV-B5, CV-B6, CV-A9, all echoviruses, enterovirus B69 (EV-B69), EV-B73, EV-B74, EV-B75, and EV-B77-107 (Adams et al. 2013; King et al. 2012). Enterovirus C members include Coxsackievirus A1 (CV-A1), CV-A11, CV-A13, CV-A17, CV-A19, CV-A20, CV-A21, CV-A22, CV-A24, EV-C95, EV-C96, EV-C99, EV-C102, EV-C104, EV-C105, EV-C109, EV-C113, EV-C116, EV-C117, EV-C118 and Polioviruses (PV1-3) (Adams et al. 2013; King et al. 2012; Brown et al. 2003) while Enterovirus D comprises Enterovirus D68 (EV-D68), EV-D70, EV-D94 and EV-D111 types (Adams et al. 2013; King et al. 2012). To date, over 100 antigenically distinct human enterovirus (HEV) types have been described (Chiang et al. 2012).

The conventional method for typing EV is by isolation in cell culture followed by neutralization tests using pooled antisera (Kiang et al. 2009; Nasri et al. 2007). However, neutralization assays are labor-intensive and often result in untypeable strains (Chiang et al. 2012; Nasri et al. 2007). Presently, typing of EV is often based on reverse transcription-PCR followed by sequencing (Kumar et al. 2013; Nasri et al. 2007; Perera et al. 2010; Tan et al. 2011; Casas et al. 2001; Nix et al. 2006; Kroneman et al. 2011). Different genomic regions, especially those encoding viral structural proteins are usually targeted (Perera et al. 2010). The enterovirus genome is approximately 7.5 kb and is composed of a single open reading frame (ORF) flanked by 5′ and 3′ untranslated regions (UTRs) (Hu et al. 2014; Solomon et al. 2010). The ORF encodes a single polyprotein that is cleaved by viral proteases to yield structural proteins VP1–VP4 and non-structural proteins 2A, 2B, 2C, 3A, 3B, 3C and 3D (Hu et al. 2014; Solomon et al. 2010). Among the structural proteins, the VP1 is the major surface-accessible viral capsid and contains a type-specific antigenic neutralization site, the BC-loop (Reimann et al. 1991; Norder et al. 2003). Genotyping of enteroviruses based on the VP1 gene has been shown to correlate well with those made by antigenic properties (Oberste et al. 1999, 2003; Nasri et al. 2007; Pallansch and Roos 2007).

Typing of EV is essential as it can provide vital information for establishment of temporal patterns of circulation of different types, detection of newly emerging or unidentified types, guidance to outbreak investigation and disease association studies, guidance to development of new diagnostics/therapies, and augmentation of poliomyelitis surveillance (Khetsuriani et al. 2006; Chiang et al. 2012; Kumar et al. 2013; Nasri et al. 2007). Here, we report on the diversity of HEV types isolated in a pediatric population in Kenya between 2008 and 2011 by sequencing a portion of the VP1 gene.

## Methods

### Study sites, inclusion criteria, clinical parameters and virus isolation

The HEV isolates (n = 200) used in this study were first propagated in culture and presence of the virus confirmed by indirect fluorescent antibody (IDFA) test using Amplifluor^®^ ID Pan-Enterovirus Detection Kit (Millipore Corporation, USA) according to the manufacturer’s instructions. They were retrieved from archives of the respiratory virus surveillance program in the Department of Emerging Infectious Diseases (DEID) of the United States Army Medical Research Directorate-Kenya in collaboration with the Kenya Medical Research Institute (KEMRI). The program surveillance network was selected to include disparate geographic regions and population demographics across the country. The inclusion criteria consisted of being an outpatient, ≥2 months of age, and having influenza-like-illness (ILI) symptoms as defined by the WHO Global Surveillance Manual on Influenza (WHO 2012). Demographic information including age, sex, occupation, and workplace and residence history were ascertained for all participants. Clinical parameters and symptoms including recent history of ILI, cough, difficulty in breathing, chills, sore throat, muscle aches, retro-orbital pain, malaise, vomiting, neurological symptoms, abdominal pain, nasal stuffiness, runny nose, sputum production, headache, joint pain, fatigue, diarrhea, and bleeding were documented. Nasopharyngeal specimens were collected and viruses isolated using rhabdomyosarcoma (RD) cells (ATCC^®^ CCL-136™) prepared in culture tubes (Nunc, Denmark). Each culture tube containing RD cells in Dulbecco’s minimum essential medium (Life Technologies, USA) was infected with 100 µl of specimen and incubated at 33 °C with 5 % CO_2_ until the appearance of cytopathic effects (CPEs) or 14 days post-infection with no observable CPEs.

### Ethics statement

The study objectives were verbally explained to the parents/guardians/next of kin and consent obtained before sample collection. After verbal explanation of the objectives by study personnel, the parents/guardians/next of kin were given time to read and understand the questionnaire in a language they understood. Then, the parent/guardian/next of kin was presented with duplicate written informed consent forms allowing for collection and testing of a specimen. An independent and trusted witness read the consent forms for those who were illiterate. The witness guided them through the consent process and in appending their signatures/thumbprint on the consent form. Patients were allowed to withdraw consent at any point during the study. Of the duplicate copies, the first copy was retained by the patient/guardian and the other copy was kept on record for regulatory review. Two ethical review boards, the Walter Reed Army Institute of Research (WRAIR) Institutional Review Board (IRB) and the Kenya Medical Research Institute (KEMRI) Ethics Review Committee (ERC), approved this study and consent procedure under protocol approvals WRAIR#1991 and KEMRI SSC#2383 respectively.

### Partial VP1 reverse transcription-PCR and sequencing

Viral RNA was extracted from infected culture supernatants with a QIAamp Viral Mini Kit (Qiagen, Inc., USA), according to the manufacturer’s instructions. Partial VP1 gene (3′-end of VP1 gene) was amplified by RT-PCR using primers 292 (5′-MIGCIGYIGARACNGG-3′, position: 2612–2627) and 222 (5′-CICCIGGIGGIAYRWACAT-3′, position: 2969–2951) as previously described (Oberste et al. 2006; Vignuzzi et al. 2005). PCR amplicons (~350 bp) were analyzed by electrophoresis using 1.0 % Agarose gels (Sigma-Aldrich Co., USA), stained in ethidium bromide (0.5 μg/ml) and visualized using the Alpha Imager (Alpha Innotech, USA). Amplicons were purified using Exonuclease I/Shrimp Alkaline Phosphatase (ExoSap-IT) enzyme (Affymetrix, USA) and sequenced directly in both directions using the RT-PCR primers. Sequencing was performed using Big Dye Terminator Cycle sequencing kit v3.1 (Applied Biosystems, USA) and analyzed using the automated 3500xL Genetic Analyzer (Applied Biosystems, USA) according to the manufacturer’s instructions.

### Sequence analysis

VP1 DNA nucleotide sequence fragments were edited and assembled into Contigs (~350 nucleotides) using DNA baser version 3.2 (2012). Type identity of the isolates was determined by homology analysis, using CLC genomics Workbench v6.5 software (CLC bio, Denmark). An isolate was determined to be homologous to a prototype strain if it shared ≥75 % nucleotide identity (≥85 % amino acid similarity) with the reference strain (Oberste et al. 2003). Multiple sequence alignments were performed by Muscle v3.8 software (Edgar 2004). Phylogenetic analyses based on nucleotide sequences of the VP1 region were carried out using MrBayes v3.2 software and MEGA 6.0 program (Ronquist et al. 2012; Tamura et al. 2013). The Bayesian trees generated were visualized using Fig Tree v1.4.0 software (Rambaut 2009).

### GenBank accession numbers

Nucleotide sequences of the partial VP1 gene of EV isolates reported in this study are available in the GenBank under accession numbers: KJ472833 to KJ472893.

### Prototype strains

The EV prototype strains whose VP1 nucleotide sequences were used in this study were retrieved from the GenBank database under accession numbers: AB426608 to AB426609; AJ493062; AY036579; AY208120; AY421760 to AY421769; AY302539 to AY302556; AY302560; AY426531; AY556057; AY556070; AY697458 to AY697461; AY919484; AF029859; AF039205; AF081311; AF081485; AF083069; AF114383; AF162711; AF241359; AF317694; AF499635 to AF499637; AF499639; AF499641 to AF499643; AF546702; EF555644 to EF555645; AY843297 to AY843299; AY843300; AY843301; AY843303-AY843308; D00627; DQ201177; DQ902712; DQ916377; EF127244; EF015886; EU840733; GQ865517; JQ446368; JQ768163; JX514942-JX514943; JX417822; K01392; KC787153; KF312882; KF385945; M12197; M16560; M88483; U05876; U22521; V01149; X05690; X79047; X80059; X84981 (source: http://www.picornaviridae.com/enterovirus/enterovirus.htm).

## Results

Overall, 187 (93.5 %) of the archived isolates propagated in culture were confirmed positive for EV by IDFA. Of these, 124 (66.3 %) RNA extracts amplified successfully by RT-PCR. Upon nucleotide sequencing, 85 (68.5 %) RT-PCR amplicons yielded clean and readable sequences. Preliminary BLASTn search analyses revealed that 61 were Enterovirus species while 24 were Rhinoviruses. Among the patients from whom enterovirus amplicons were detected; 35 (57.4 %) were males while 26 (42.6 %) females, giving a gender ratio of 1.4:1. The age of patients ranged from 2 months to 7 years. Infections were more common in children aged <3 years (40/61, 65.6 %) than those aged ≥3 years (34.4 %). The most common clinical manifestations included cough (100.0 %), runny nose (91.8 %) and stuffy nose (47.5 %). Other symptoms included vomiting (29.5 %), malaise (27.9 %), fatigue (23.0 %), chills (21.3 %), diarrhea (19.7 %), difficulty in breathing (16.4 %), headache (11.5 %), abdominal pain (11.5 %) and sore throat, neurological conditions and joint pains (<1.0 %).

**Fig. 1:**
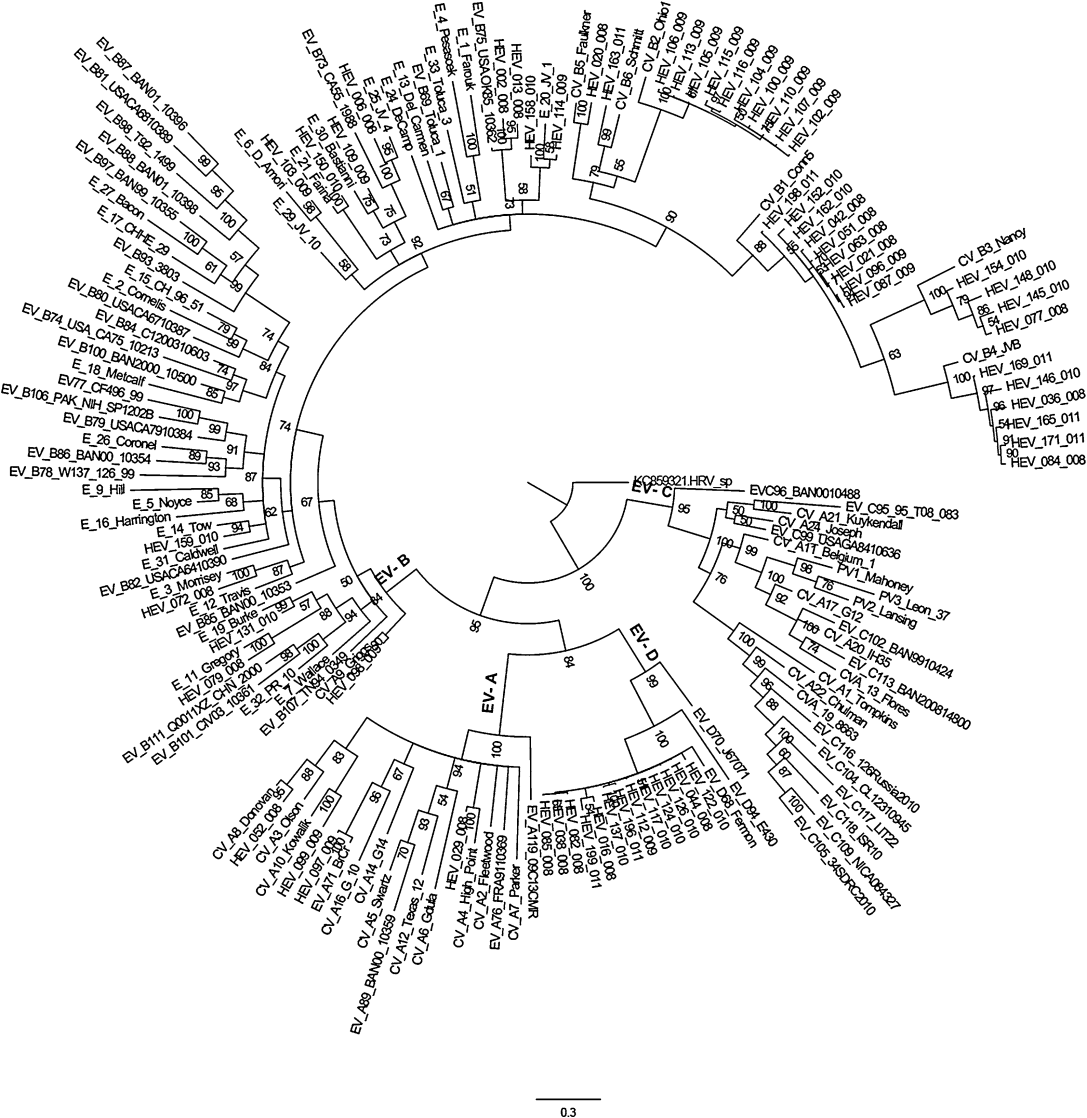
Bayesian phylogenetic tree based on nucleotide sequences of the 3′-end of the VP1 capsid region of human enterovirus strains. The tree was estimated using MrBayes 3.2 with a general time-reversible (GTR) substitution model. Posterior probabilities support values are shown on each node. The *scale bar* indicates number of nucleotide substitutions per site. *EV-A* Enterovirus A, *EV-B* Enterovirus B, *EV-C* Enterovirus C s, *EV-D* Enterovirus D species. Kenyan strains are designated starting with HEV. The out-group is rhinovirus

**Fig. 2:**
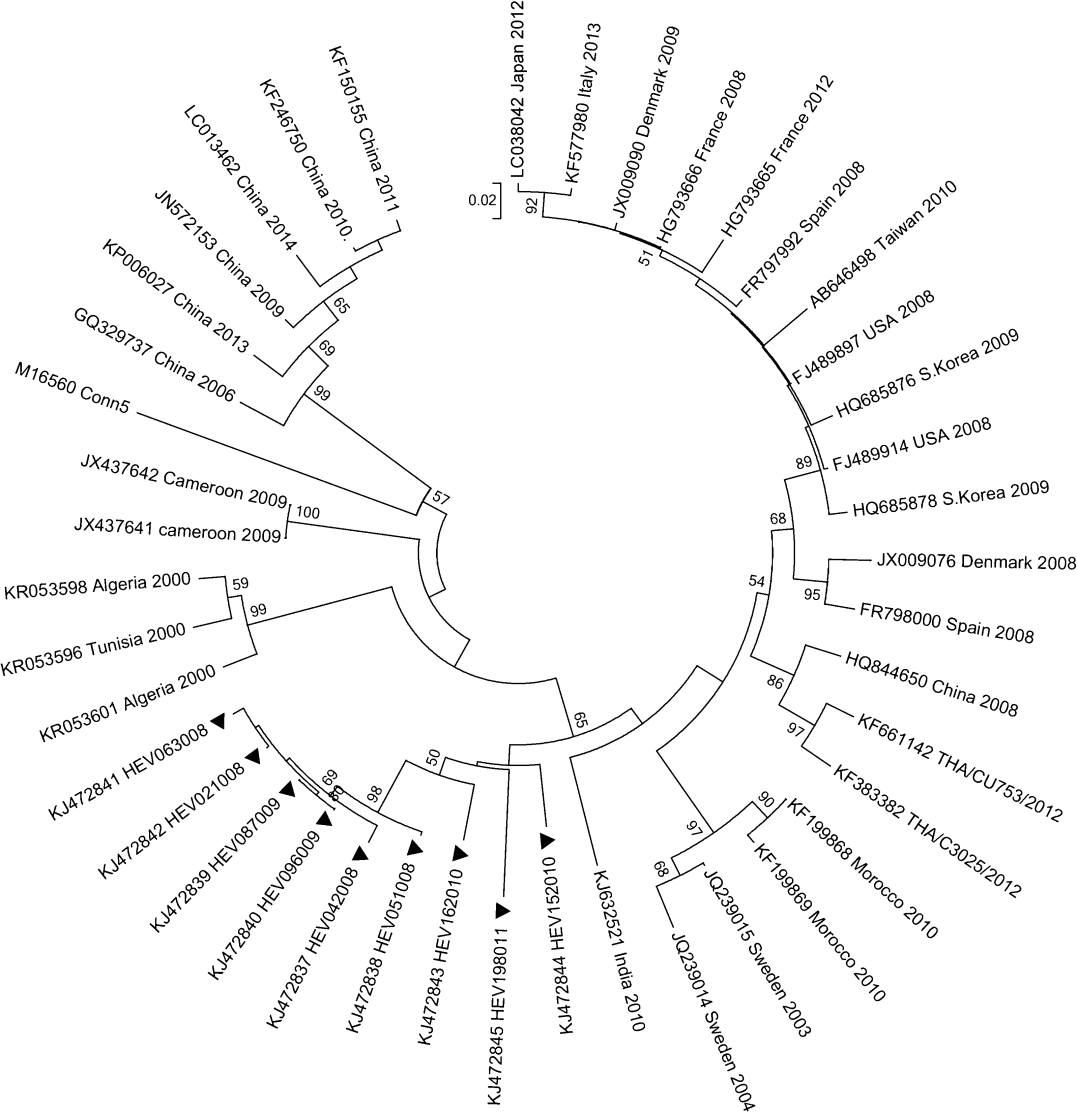
Phylogenetic analysis based on nucleotide sequences of the 3′-end of the VP1 gene of CV-B1 strains. The tree was generated by MEGA 6.0 using the neighbor-joining method. The *filled triangles* indicate the strains obtained in this study

**Fig. 3:**
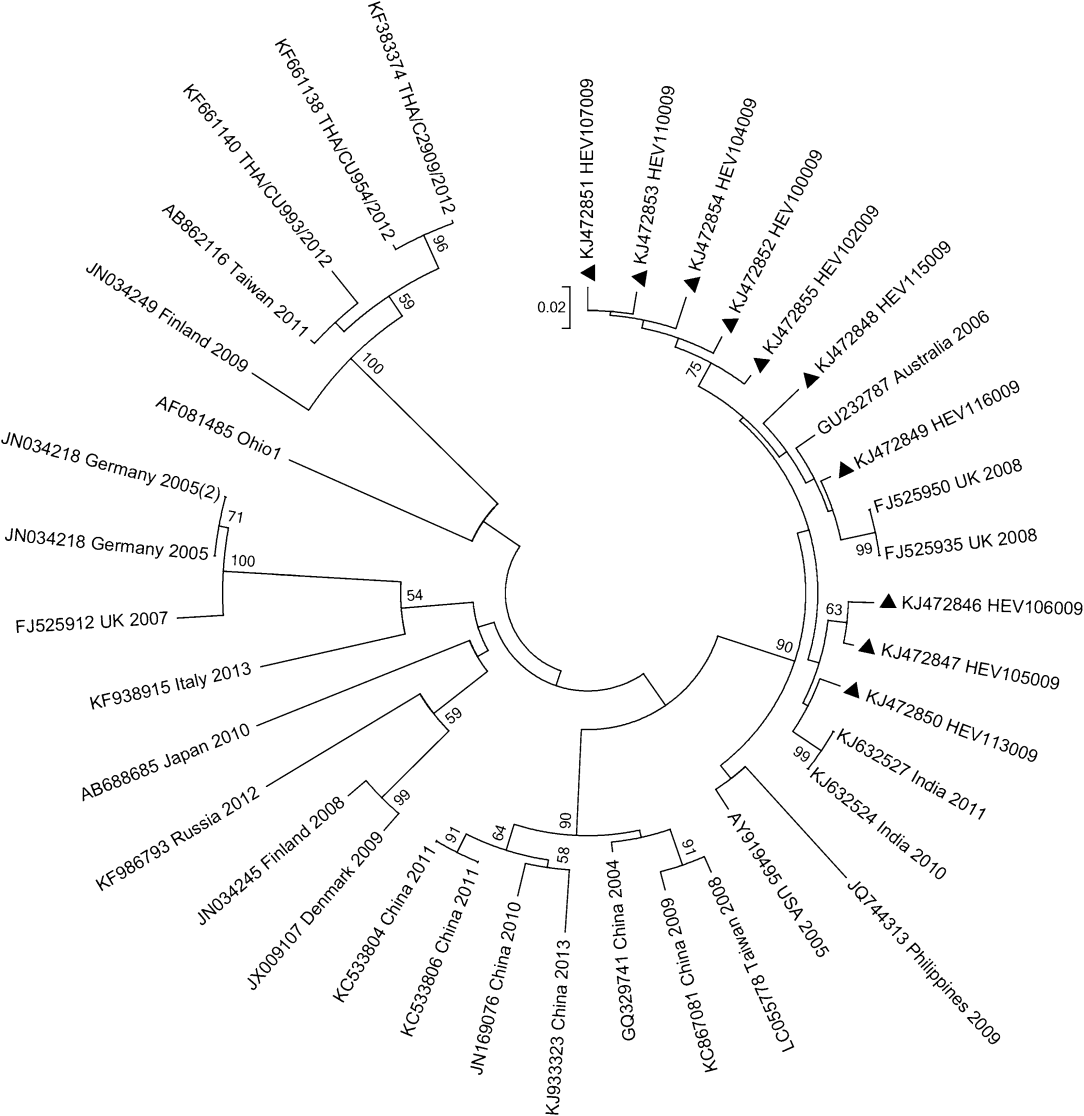
Phylogenetic analysis of CV-B2 strains. Nucleotide sequences of the 3′-end of the VP1 gene were analyzed by the neighbor-joining methods and the tree generated by MEGA 6.0. The *filled triangles* indicate the strains obtained in this study

**Fig. 4:**
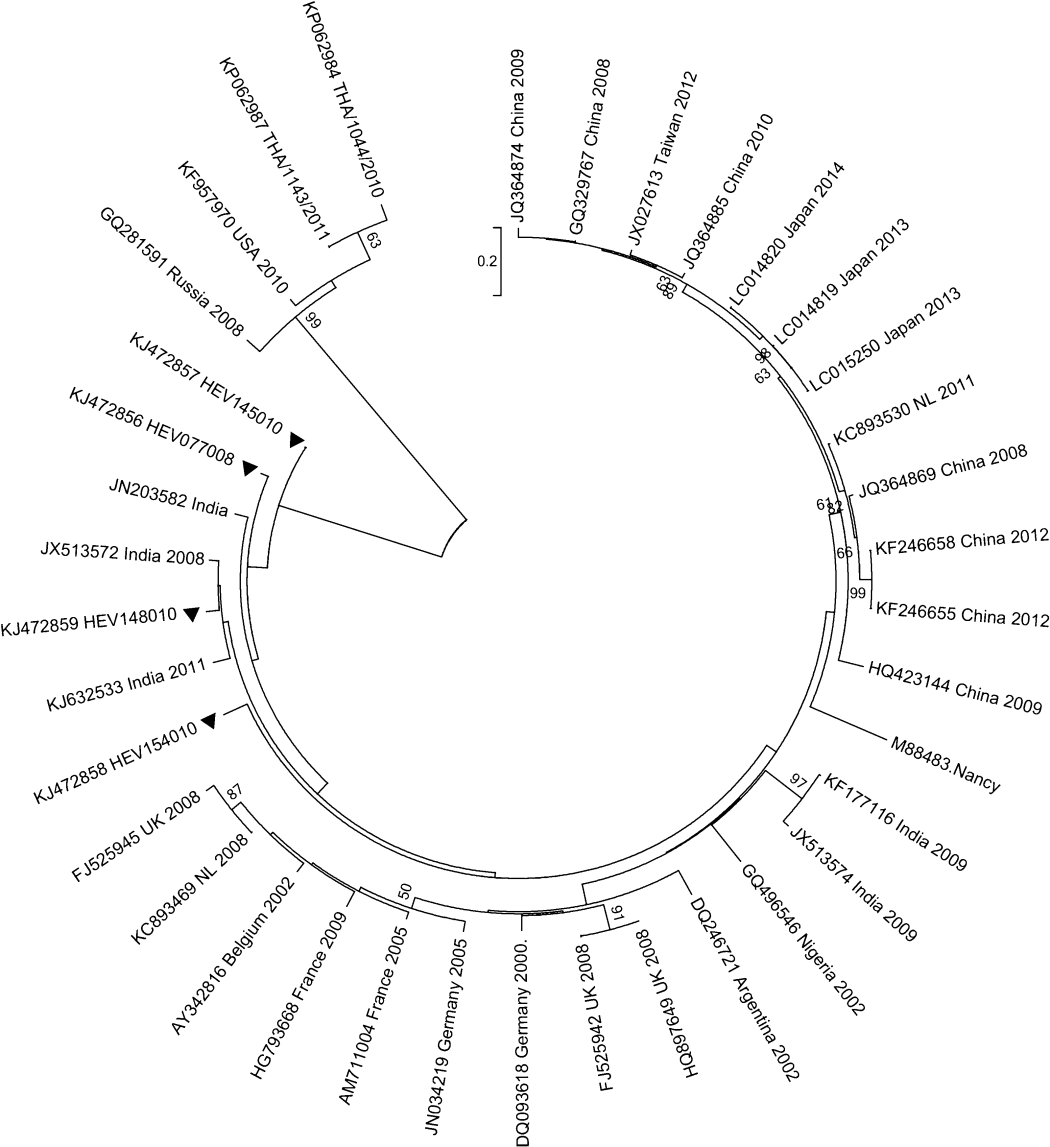
Phylogenetic relationships of CV-B3 strains. The tree was constructed by MEGA 6.0 based on nucleotide sequences of the 3′-end of the VP1 gene, using the neighbor-joining method. The *filled triangles* indicate the stains obtained in this study

**Fig. 5:**
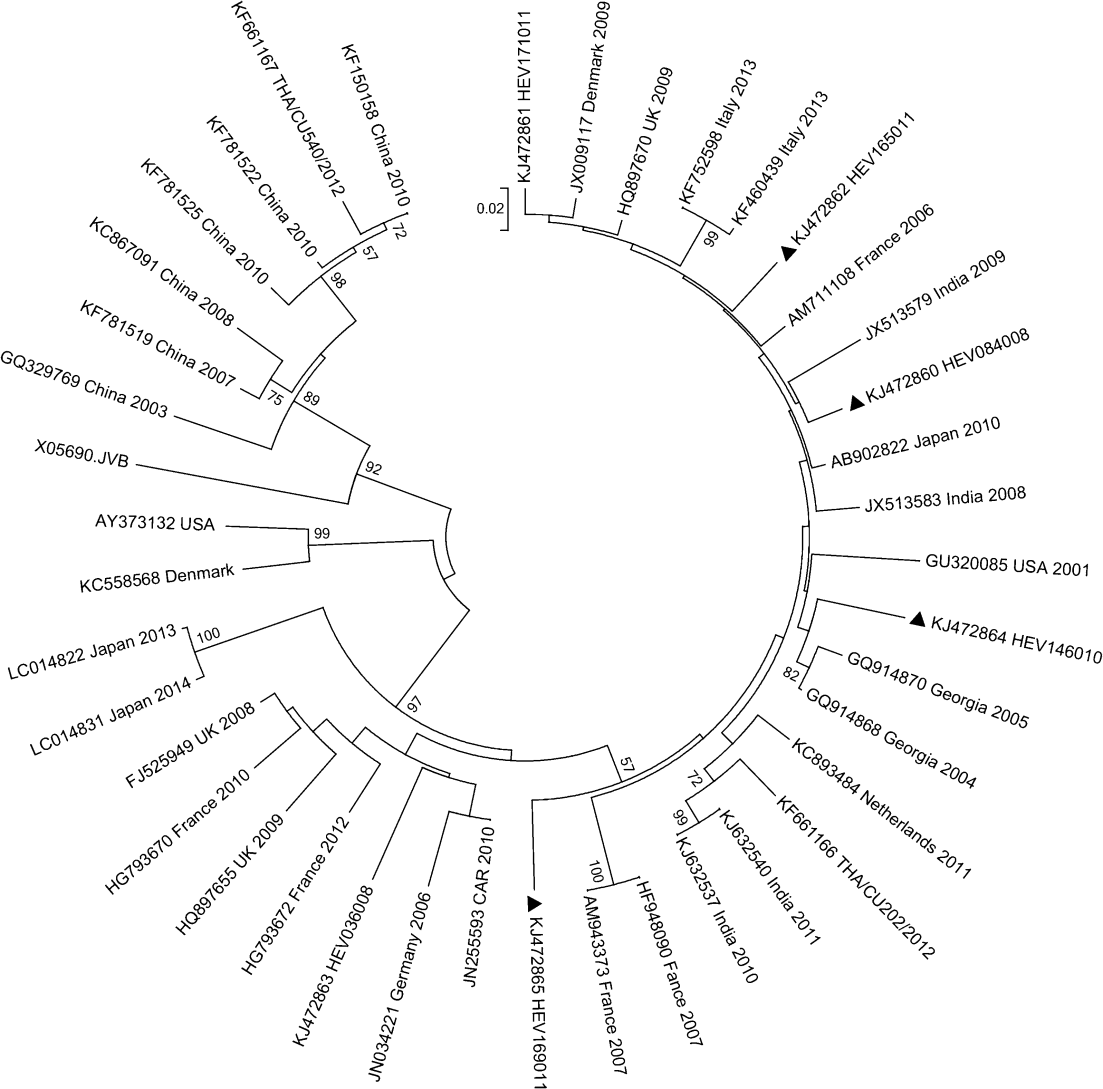
Phylogenetic analysis of CV-B4 strains, based on nucleotide sequences of the 3′-end of the VP1 gene. The tree was constructed by using MEGA 6.0, using the neighbor-joining method. The *filled triangles* indicate the strains obtained in this study

**Fig. 6:**
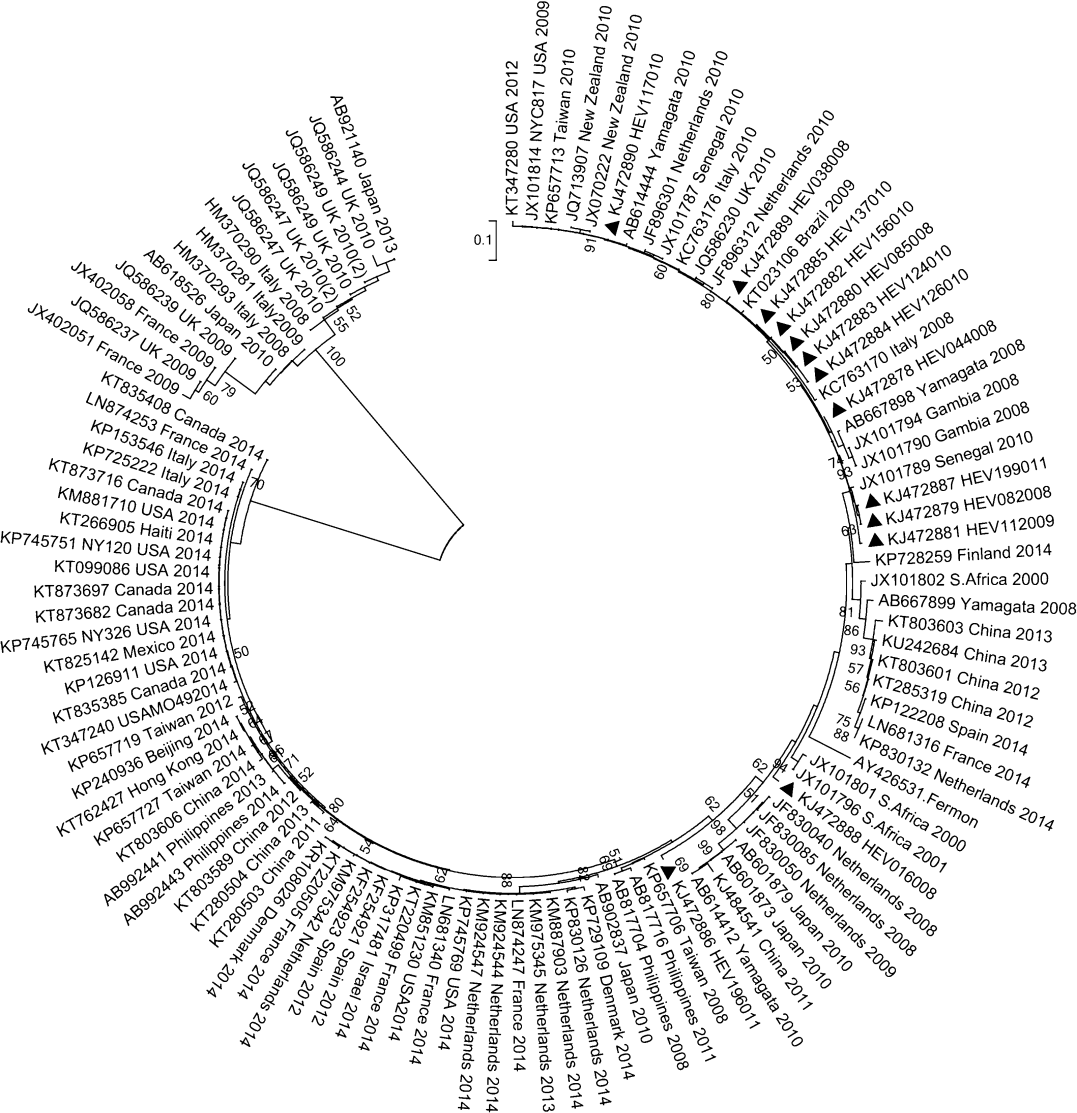
Phylogenetic relationships of EV-D68 strains, based on nucleotide sequences of the 3′-end of the VP1 gene. The tree was constructed using the neighbor-joining method implemented in MEGA 6.0. The *filled triangles* indicate the strains obtained in this study

Homology analyses based on 3′-end of the VP1 gene showed that majority of EV types detected in Kenya (except echovirus 11 strain; GeneBank accession number KJ472870) shared ≥75.0 % (nucleotide) or ≥85.0 % amino acid sequence identities with homotypic prototype strains (Table 1). The Kenyan echovirus 11 isolate exhibited a slightly lower nucleotide sequence identity (74.02 %) when compared to echovirus-11 prototype strain (Gregory) but higher nucleotide sequence identities (89–90 %) with recently isolated echovirus-11 strains with GenBank accession numbers GU393825 and JQ744302, respectively. Majority of the Kenyan isolates belonged to Enterovirus B species (n = 44) followed by Enterovirus D (n = 13) and Enterovirus A (n = 4). None of the isolates belonged to Enterovirus C species (Table 1). Confirmation of type identities assigned to the isolates by pair-wise alignment obtained using phylogeny of the Kenyan isolates and those of HEV prototype strains revealed separation of the sequences into four distinct groups, delineating Enterovirus A, Enterovirus B, Enterovirus C and Enterovirus D species (Fig. 1). All the Kenyan isolates clustered closely with respective homotypic prototypes, supported by high posterior probability nodal values of 80.0–100.0 %. Majority of the isolates (n = 44) clustered with Enterovirus B species prototype strains followed by Enterovirus D (n = 13) and Enterovirus A (n = 4) (Fig. 1). These results corroborated those determined by homology analysis. Moreover, phylogenetic analyses of the most frequently detected EV types with sequences of homotypic reference strains sampled from GenBank revealed segregation of Kenyan CV-B1 isolates from the global strains used in the study (Fig. 2). The Kenyan CV-B2 strains were closely related to those isolated in countries such as Australia (2006), the United Kingdom (2008) and India (2010–2011) (Fig. 3). All the Kenyan CV-B3 isolates clustered closely with Indian strains isolated between 2008 and 2009 (Fig. 4) while the CV-B4 generally appeared interspersed among sequences of homotypic strains that circulated in Europe, Asia and the United States of America (Fig. 5). The Kenyan EV-D68 isolates clustered together mostly with sequences of EV-D68 strains which circulated in the world between 2008 and 2010 (Fig. 6).

**Table 1:** Comparison of the partial VP1 sequences of EV types detected in Kenya with those of homotypic prototypes

EV type (n)	Sequence identities to homotypic EV prototype strains	
	% Nucleotide	% Amino acid
CV-A4 (1)	85.39	100
CV-A8 (1)	88.52	98.02
CV-A10 (1)	75.03	92.16
EV-D71 (1)	97.75	99.03
CV-A9 (1)	90.32	96.43
EV-D75 (2)	85.71–87.1	96.09–98.32
CV-B1 (9)	77.03–80.92	95.12
CV-B2 (10)	81.36–83.18	96.47–97.65
CV-B3 (4)	75.2–77.56	90.12–92.59
CV-B4 (6)	78.34–82.95	92.86–100
CV-B5 (1)	85.51	93.98
CV-B6 (1)	75.35	93.98
E-3 (1)	75.45	90.48
E-6 (1)	80.65	95.24
E-11 (1)	74.02	89.29
E-14 (1)	79.55	92.94
E-19 (1)	79.82	98.31
E-20 (2)	80.65–82.49	94.05–95.24
E-21 (1)	90.91	96.47
E-25 (1)	81.57	95.24
E-30 (1)	79.09	91.76
EV-D68 (13)	85.94–87.5	84.89–90.48

## Discussion

This paper outlines the diversity of HEV that circulated in the Kenyan pediatric population between 2008 and 2011. The multiplicity and frequency of the types reported were determined through homology and phylogenetic analyses of sequences of the partial VP1 region, relative to prototype strains. Overall, 22 different EV types belonging to three of the seven known HEV species were identified. The bulk (72.0 %) of the types belonged to Enterovirus B species. This corroborates findings of Enterovirus diversity from elsewhere (Harvala et al. 2014; Tan et al. 2011; Trallero et al. 2010; Bessaud et al. 2012). 21.3 % belonged to Enterovirus D species while 6.5 % were Enterovirus A. None of the types belonged to Enterovirus C. None to low detection levels (ranging from 0.0 to <5.0 %) of members of Enterovirus C species is not peculiar to Kenya but have been reported elsewhere (Khetsuriani et al. 2006; Tan et al. 2011; Bahri et al. 2005; Roth et al. 2007; Tseng et al. 2007). The high detection levels of Enterovirus B types is attributed to replication efficiency these viruses have in cell culture compared to those of Enterovirus A and Enterovirus C species and other numbered EV types (Tan et al. 2011; Bryden 1992; Heim 2005; Kargar et al. 2009; Schmidt et al. 1975). Primers specific to enterovirus VP1 gene may fail to amplify some members of Enterovirus A and D species (Nasri et al. 2007). Moreover, the absence of EV-C and low detection levels of EV-A viruses may be attributed to the use of only one cell line (RD cell line) during the isolation process (Rakoto-Andrianarivelo et al. 2005). The failure of some samples to amplify by RT-PCR and sequence properly may be attributed to mixed enterovirus types (Oberste et al. 2006; Apostol et al. 2012) in the culture supernatants or other members of *Enterovirus genus* such as Rhinovirus species. Indeed, preliminary BLASTn search analyses in this work showed that 28.2 % of the readable sequences generated were Rhinoviruses. Besides, the pan-enterovirus primers (292/222) used here are only able to amplify some members of Rhinovirus species (Oberste and Pallansch 2003).

The most frequently detected type strains were Enterovirus-D68 followed by Coxsackievirus B2 (CV-B2), CV-B1, CV-B4, and CV-B3. Notably, enterovirus-D68 was the only species-D type detected in this study. This was not surprising as reports of increased worldwide circulation of enterovirus-D68 viruses in recent times have been published (Imamura et al. 2011; Kaida et al. 2011; Rahamat-Langendoen et al. 2011; Tokarz et al. 2012; Opanda et al. 2014). Enterovirus-68 has primarily been associated with respiratory disease (Imamura et al. 2011; Tokarz et al. 2012; Hasegawa et al. 2011; Meijer et al. 2012; Piralla et al. 2014). Besides, enterovirus-B viruses are a significant cause of infections in children (Kumar et al. 2013). CV-B1, CV-B2, CV-B3 and CV-B4 type strains have been frequently implicated with outbreaks of aseptic meningitis in young infants (Khetsuriani et al. 2006; Tao et al. 2012; Wong et al. 2011; Verma et al. 2009; Wikswo et al. 2009; Rezig et al. 2004). The enterovirus A types detected in this work (i.e. EV-A71, CV-A8 and CV-A10) have more frequently been associated with sporadic outbreaks of hand, foot and mouth disease (Tan et al. 2011; Zhu et al. 2013; Chen et al. 2016; Lu et al. 2012). The Kenyan Echovirus 11 isolate appeared to be genetically different from the prototype strain (Gregory) but closely similar to sequences of contemporaneous E-11 strains isolated in Azerbaijan (2006) and Philippines (2009), suggesting evolutionary divergence from the prototype strain. Phylogenetic relationships of the most frequently detected EV types showed that the Kenyan CV-B1 isolates were genetically different from the global strains. CV-B2, CV-B3, CV-B4 and EV-D68 isolates were generally closely related to other strains detected from different countries, suggesting worldwide circulation of these viruses. The clustering of the Kenyan EV-D68 isolates mostly with sequences of global strains obtained in other countries between 2008 and 2010 in contrast to those isolated in recent years may be attributed to high genomic variability associated with RNA viruses, including enteroviruses (Apostol et al. 2012; Holland et al. 1982).

Majority of the patients from whom the viruses were detected presented with symptoms associated with mild respiratory illnesses such as cough (100.0 %) and runny nose (91.8 %). Other clinical manifestations were also recorded. However clinical diagnoses were not provided by the Clinicians owing to study design. The sex ratio of the patients was 1.4:1 for male (57.4 %) to female (42.6 %). This mirrors findings from previous studies (Tao et al. 2014), plausibly suggesting higher exposure of male children to enterovirus infection compared to female.

This work had some drawbacks. Being a retrospective study relying on isolates, some enteroviruses may have failed to grow in culture due to loss of viability during storage. In addition, culture of these viruses in one type of cell line may have led to selective growth of some types and not others. These two shortcomings may have introduced bias in the diversity of EV types reported. Furthermore, the lone utilization of the partial VP1 region to detect the viruses may have led to non-detection of some enteroviruses (Perera et al. 2010; Oberste et al. 2006). Despite these limitations, we have demonstrated circulation and considerable diversity of enterovirus strains within the Kenyan pediatric population between 2008 and 2011. In future, full genome studies will augment our findings.

## References

[CR2] Adams M, King A, Carstens E (2013). Ratification vote on taxonomic proposals to the International Committee on Taxonomy of Viruses (2013). Arch Virol.

[CR3] Adams M, Lefkowitz E, King A, Carstens E (2014). Ratification vote on taxonomic proposals to the International Committee on Taxonomy of Viruses (2014). Arch Virol.

[CR4] Adams MJ, Lefkowitz EJ, King AMQ, Bamford DH, Breitbart M, Davison AJ, Ghabrial SA, Gorbalenya AE, Knowles NJ, Krell P, Lavigne R, Prangishvili D, Sanfaçon H, Siddell SG, Simmonds P, Carstens EB (2015). Ratification vote on taxonomic proposals to the International Committee on Taxonomy of Viruses (2015). Arch Virol.

[CR5] Apostol LNG, Imagawa T, Suzuki A, Masago Y, Lupisan S (2012). Genetic diversity and molecular characterization of enteroviruses from sewage-polluted urban and rural rivers in the Philippines. Virus Genes.

[CR6] Bahri O, Rezig D, Nejma-Oueslati BB, Yahia AB, Sassi JB (2005). Enteroviruses in Tunisia: virological surveillance over 12 years (1992–2003). J Med Microbiol.

[CR7] Bessaud M, Pillet S, Ibrahim W, Joffret M-L, Pozzetto B (2012). Molecular characterization of human enteroviruses in the Central African Republic: uncovering wide diversity and identification of a new human enterovirus A71 genogroup. J Clin Microbiol.

[CR8] Brown BA, Pallansch MA (1995). Complete nucleotide sequence of enterovirus 71 is distinct from poliovirus. Virus Res.

[CR9] Brown B, Oberste MS, Maher K, Pallansch MA (2003). Complete genomic sequencing shows that polioviruses and members of human enterovirus species C are closely related in the noncapsid coding region. J Virol.

[CR10] Bryden A (1992). Isolation of enteroviruses and adenoviruses in continuous simian cell lines. Med Lab Sci.

[CR11] Casas I, Palacios G, Trallero G, Cisterna D, Freire M (2001). Molecular characterization of human enteroviruses in clinical samples: comparison between VP2, VP1, and RNA polymerase regions using RT nested PCR assays and direct sequencing of products. J Med Virol.

[CR12] Chen L, Yang H, Wang C, Yao X-J, Zhang H-L (2016). Genomic characteristics of coxsackievirus A8 strains associated with hand, foot, and mouth disease and herpangina. Arch Virol.

[CR13] Chiang P-S, Huang M-L, Luo S-T, Lin T-Y, Tsao K-C (2012). Comparing molecular methods for early detection and serotyping of enteroviruses in throat swabs of pediatric patients. PLoS One.

[CR1] DNA-Baser Assembler v3. 2 (2012) Heracle BioSoft SRL Romania

[CR14] Edgar RC (2004). MUSCLE: multiple sequence alignment with high accuracy and high throughput. Nucleic Acids Res.

[CR15] Harvala H, Calvert J, Van Nguyen D, Clasper L, Gadsby N (2014). Comparison of diagnostic clinical samples and environmental sampling for enterovirus and parechovirus surveillance in Scotland, 2010 to 2012. Euro Surveill.

[CR16] Hasegawa S, Hirano R, Okamoto-Nakagawa R, Ichiyama T, Shirabe K (2011). Enterovirus 68 infection in children with asthma attacks: virus-induced asthma in Japanese children. Allergy.

[CR17] Heim A (2005). From poliovirus surveillance to enterovirus surveillance: a complete picture?. J Med Microbiol.

[CR18] Holland J, Spindler K, Horodyski F, Grabau E, Nichol S (1982). Rapid evolution of RNA genomes. Science.

[CR19] Hu Y, Yang F, Du J, Dong J, Zhang T (2011). Complete genome analysis of coxsackievirus A2, A4, A5, and A10 strains isolated from hand, foot, and mouth disease patients in China revealing frequent recombination of human enterovirus A. J Clin Microbiol.

[CR20] Hu L, Zhang Y, Hong M, Zhu S, Yan D (2014). Phylogenetic evidence for multiple intertypic recombinations in enterovirus B81 strains isolated in Tibet. China. Sci Rep.

[CR21] Imamura T, Fuji N, Suzuki A, Tamaki R, Saito M (2011). Enterovirus 68 among children with severe acute respiratory infection, the Philippines. Emerg Infect Dis.

[CR22] Jacques J, Moret H, Minette D, Lévêque N, Jovenin N (2008). Epidemiological, molecular, and clinical features of enterovirus respiratory infections in French children between 1999 and 2005. J Clin Microbiol.

[CR23] Kaida A, Kubo H, J-i Sekiguchi, Kohdera U, Togawa M (2011). Enterovirus 68 in children with acute respiratory tract infections, Osaka, Japan. Emerg Infect Dis.

[CR24] Kargar M, Sadeghipour S, Nategh R (2009). Environmental surveillance of non-polio enteroviruses in Iran. Virol J.

[CR25] Khetsuriani N, LaMonte-Fowlkes A, Oberst S, Pallansch MA (2006). Enterovirus surveillance—United States, 1970–2005. MMWR Surveill Summ.

[CR26] Kiang D, Newbower EC, Yeh E, Wold L, Chen L (2009). An algorithm for the typing of enteroviruses and correlation to serotyping by viral neutralization. J Clin Virol.

[CR27] King AM, Adams MJ, Lefkowitz EJ, Carstens EB (2012) Virus taxonomy: classification and nomenclature of viruses. In: Ninth report of the International Committee on Taxonomy of Viruses. Elsevier

[CR28] Kroneman A, Vennema H, Deforche K, Avoort H, Penaranda S (2011). An automated genotyping tool for enteroviruses and noroviruses. J Clin Virol.

[CR29] Kumar A, Shukla D, Srivastava S, Idris MZ, Dhole TN (2013). High frequency of enterovirus serotype circulation in a densely populated area of India. J Infect Dev Ctries.

[CR30] Linsuwanon P, Puenpa J, Suwannakarn K, Auksornkitti V, Vichiwattana P (2012). Molecular epidemiology and evolution of human enterovirus serotype 68 in Thailand, 2006–2011. PLoS One.

[CR31] Lu Q-B, Zhang X-A, Wo Y, Xu H-M, Li X-J (2012). Circulation of Coxsackievirus A10 and A6 in hand-foot-mouth disease in China, 2009–2011. PLoS One.

[CR32] Meijer A, van der Sanden S, Snijders BE, Jaramillo-Gutierrez G, Bont L (2012). Emergence and epidemic occurrence of enterovirus 68 respiratory infections in The Netherlands in 2010. Virology.

[CR33] Nasri D, Bouslama L, Pillet S, Bourlet T, Aouni M (2007). Basic rationale, current methods and future directions for molecular typing of human enterovirus. Expert Rev Mol Diagn.

[CR34] Nasri D, Bouslama L, Omar S, Saoudin H, Bourlet T (2007). Typing of human enterovirus by partial sequencing of VP2. J Clin Microbiol.

[CR35] Nix WA, Oberste MS, Pallansch MA (2006). Sensitive, seminested PCR amplification of VP1 sequences for direct identification of all enterovirus serotypes from original clinical specimens. J Clin Microbiol.

[CR36] Norder H, Bjerregaard L, Magnius L, Lina B, Aymard M (2003). Sequencing of ‘untypable’ enteroviruses reveals two new types, EV-77 and EV-78, within human enterovirus type B and substitutions in the BC loop of the VP1 protein for known types. J Gen Virol.

[CR37] Oberste MS, Pallansch MA (2003). Establishing evidence for enterovirus infection in chronic disease. Ann N Y Acad Sci.

[CR38] Oberste MS, Maher K, Kilpatrick DR, Pallansch MA (1999). Molecular evolution of the human enteroviruses: correlation of serotype with VP1 sequence and application to picornavirus classification. J Virol.

[CR39] Oberste MS, Nix WA, Maher K, Pallansch MA (2003). Improved molecular identification of enteroviruses by RT-PCR and amplicon sequencing. J Clin Virol.

[CR40] Oberste MS, Peñaranda S, Maher K, Pallansch MA (2004). Complete genome sequences of all members of the species Human enterovirus A. J Gen Virol.

[CR41] Oberste MS, Maher K, Michele SM, Belliot G, Uddin M (2005). Enteroviruses 76, 89, 90 and 91 represent a novel group within the species Human enterovirus A. J Gen Virol.

[CR42] Oberste MS, Maher K, Williams AJ, Dybdahl-Sissoko N, Brown BA (2006). Species-specific RT-PCR amplification of human enteroviruses: a tool for rapid species identification of uncharacterized enteroviruses. J Gen Virol.

[CR43] Opanda SM, Wamunyokoli F, Khamadi S, Coldren R, Bulimo WD (2014). Genetic diversity of human enterovirus 68 strains isolated in kenya using the hypervariable 3′-end of VP1 gene. PLoS One.

[CR44] Pallansch MA, Roos R, Knipe DM, Howley PM (2007). Enteroviruses: polioviruses, coxsackie viruses, echoviruses, and newer enteroviruses. Fields virology.

[CR45] Perera D, Shimizu H, Yoshida H, Van Tu P, Ishiko H (2010). A comparison of the VP1, VP2, and VP4 regions for molecular typing of human enteroviruses. J Med Virol.

[CR46] Piralla A, Girello A, Grignani M, Gozalo-Margüello M, Marchi A (2014). Phylogenetic characterization of enterovirus 68 strains in patients with respiratory syndromes in Italy. J Med Virol.

[CR47] Pöyry T, Hyypiä T, Horsnell C, Kinnunen L, Hovi T (1994). Molecular analysis of coxsackievirus A16 reveals a new genetic group of enteroviruses. Virology.

[CR48] Rahamat-Langendoen J, Riezebos-Brilman A, Borger R, van der Heide R, Brandenburg A (2011). Upsurge of human enterovirus 68 infections in patients with severe respiratory tract infections. J Clin Virol.

[CR49] Rakoto-Andrianarivelo M, Rousset D, Razafindratsimandresy R, Chevaliez S, Guillot S (2005). High frequency of human enterovirus species C circulation in Madagascar. J Clin Microbiol.

[CR50] Rambaut A (2009) FigTree v1. 4.0: Tree Figure Drawing Tool. http://tree.bio.ed.ac.uk/software/figtree/

[CR51] Reimann B-Y, Zell R, Kandolf R (1991). Mapping of a neutralizing antigenic site of Coxsackievirus B4 by construction of an antigen chimera. J Virol.

[CR52] Rezig D, Yahia AB, Abdallah HB, Bahri O, Triki H (2004). Molecular characterization of coxsackievirus B5 isolates. J Med Virol.

[CR53] Ronquist F, Teslenko M, van der Mark P, Ayres DL, Darling A (2012). MrBayes 3.2: efficient Bayesian phylogenetic inference and model choice across a large model space. Syst Biol.

[CR54] Roth B, Enders M, Arents A, Pfitzner A, Terletskaia-Ladwig E (2007). Epidemiologic aspects and laboratory features of enterovirus infections in Western Germany, 2000–2005. J Med Virol.

[CR55] Schmidt NJ, Ho HH, Lennette EH (1975). Propagation and isolation of group A coxsackieviruses in RD cells. J Clin Microbiol.

[CR56] Solomon T, Lewthwaite P, Perera D, Cardosa MJ, McMinn P (2010). Virology, epidemiology, pathogenesis, and control of enterovirus 71. Lancet Infect Dis.

[CR57] Tamura K, Stecher G, Peterson D, Filipski A, Kumar S (2013). MEGA6: molecular evolutionary genetics analysis version 6.0. Mol Biol Evol.

[CR58] Tan X, Huang X, Zhu S, Chen H, Yu Q (2011). The persistent circulation of enterovirus 71 in People’s Republic of China: causing emerging nationwide epidemics since 2008. PLoS One.

[CR59] Tan CY, Ninove L, Gaudart J, Nougairede A, Zandotti C (2011). A retrospective overview of enterovirus infection diagnosis and molecular epidemiology in the public hospitals of Marseille, France (1985–2005). PLoS One.

[CR60] Tao Z, Song Y, Li Y, Liu Y, Jiang P (2012). Coxsackievirus b3, Shandong province, china, 1990–2010. Emerg Infect Dis.

[CR61] Tao Z, Wang H, Li Y, Liu G, Xu A (2014). Molecular epidemiology of human enterovirus associated with aseptic meningitis in Shandong Province, China, 2006–2012. PLoS One.

[CR62] Tokarz R, Firth C, Madhi SA, Howie SR, Wu W (2012). Worldwide emergence of multiple clades of enterovirus 68. J Gen Virol.

[CR63] Trallero G, Avellon A, Otero A, De Miguel T, Pérez C (2010). Enteroviruses in Spain over the decade 1998–2007: virological and epidemiological studies. J Clin Virol.

[CR64] Tseng FC, Huang HC, Chi CY, Lin TL, Liu CC (2007). Epidemiological survey of enterovirus infections occurring in Taiwan between 2000 and 2005: analysis of sentinel physician surveillance data. J Med Virol.

[CR65] Verma NA, Zheng XT, Harris MU, Cadichon SB, Melin-Aldana H (2009). Outbreak of life-threatening coxsackievirus B1 myocarditis in neonates. Clin Infect Dis.

[CR66] Vignuzzi M, Stone JK, Arnold JJ, Cameron CE, Andino R (2005). Quasispecies diversity determines pathogenesis through cooperative interactions in a viral population. Nature.

[CR67] WHO (2012). WHO interim global surveillance standards for influenza.

[CR68] Wikswo ME, Khetsuriani N, Fowlkes AL, Zheng X, Peñaranda S (2009). Increased activity of Coxsackievirus B1 strains associated with severe disease among young infants in the United States, 2007–2008. Clin Infect Dis.

[CR69] Wong AH, Lau C, Cheng PK, Ng AY, Lim WW (2011). Coxsackievirus B3-associated aseptic meningitis: an emerging infection in Hong Kong. J Med Virol.

[CR70] Zhu J, Luo Z, Wang J, Xu Z, Chen H (2013). Phylogenetic analysis of enterovirus 71 circulating in Beijing, China from 2007 to 2009. PLoS One.

